# Co-designing a Program to Improve Post-stroke Sexual Rehabilitation: The Promise of Innovative Methods

**DOI:** 10.3389/fresc.2022.777897

**Published:** 2022-05-11

**Authors:** Louis-Pierre Auger, Dorra Rakia Allegue, Ernesto Morales, Aliki Thomas, Johanne Filiatrault, Brigitte Vachon, Annie Rochette

**Affiliations:** ^1^School of Rehabilitation, Faculty of Medicine, Université de Montréal, Montreal, QC, Canada; ^2^Centre for Interdisciplinary Research in Rehabilitation of Greater Montreal, Montreal, QC, Canada; ^3^Department of Rehabilitation, Université Laval, Quebec City, QC, Canada; ^4^Interdisciplinary Research Centre in Rehabilitation and Social Inclusion (CIRRIS), Quebec City, QC, Canada; ^5^School of Physical and Occupational Therapy, Faculty of Medicine and Health Sciences, McGill University, Montreal, QC, Canada; ^6^Montreal Geriatric University Institute Research Center, Montreal, QC, Canada; ^7^Montreal University Institute of Mental Health, Montreal, QC, Canada

**Keywords:** sexuality, stroke, rehabilitation, co-design, implementation, Intervention Mapping

## Abstract

**Introduction:**

Most people who sustain a stroke are likely to experience sexual difficulties during their recovery. However, few people get the opportunity to address sexuality during their rehabilitation because of factors related to the organization (e.g., culture), managers (e.g., lack of resources), clinicians (e.g., perceived lack of knowledge, skills, and comfort), and clients (e.g., taboo). A multifactorial program tailored to stakeholders' needs with various complementary interventions is needed to lead to a change of practice in post-stroke sexual rehabilitation.

**Objective:**

To co-design with stakeholders (i.e., people with stroke, partners, clinicians, managers and researchers) a theory-driven multifactorial program to improve post-stroke sexual rehabilitation services.

**Methods:**

This qualitative study will be conducted in four steps using an Intervention Mapping approach and a co-design methodology divided into four phases: (1) exploration; (2) co-design; (3) validation; and (4) development. Persons with stroke, partners, clinicians and managers from five distinct stroke rehabilitation centres in the province of Quebec (Canada), and researchers will be recruited to either participate in an advisory committee or working groups throughout the study. A combination of contributions from three different types of groups (advisory group, Lego® groups, work groups) will be used for data collection. Qualitative data analysis will first be realized by two independent reviewers using the Theoretical Domains Framework, and preliminary results of analysis will be validated with the advisory and working groups.

**Conclusion:**

This study will lead to the co-design of the first theory-driven program intended to optimize post-stroke sexual rehabilitation services.

## Introduction

Stroke is the second leading cause of disability around the world (1) and can result in various consequences impacting the person, their environment, and their activities (2). Persons with stroke are likely to experience restricted participation in meaningful activities and roles because of impairments, such as hemiplegia, aphasia, cognitive impairments, or mood disorders (3, 4). These impairments may require a variety of activity adaptations and environmental modifications to maintain or improve participation (5, 6). Persons with stroke report that balance and walking difficulties, fatigue, upper limb dysfunction, and speech difficulties are among the main priorities that must be addressed in rehabilitation (7, 8). Sexuality is among other priority domains for persons with stroke (7, 9) and is part of the International Classification of Functioning, Disability and Health (ICF) core set for stroke as a relevant domain to be addressed (2).

### Sexuality After a Stroke

According to the World Health Organization, sexuality is “a central aspect of being human throughout life and encompasses sex, gender identities and roles, sexual orientation, eroticism, pleasure, intimacy and reproduction” (10). Stroke can lead to multiple difficulties related to sexuality, either because of the location and/or severity of the cerebral lesion or in relation to consequences of the stroke (11, 12). In fact, more than 50% of persons with stroke are likely to experience difficulties influencing their participation and satisfaction related to sexual activities and their partners' (13–15).

However, few persons with stroke get the opportunity to address sexuality during their rehabilitation (16, 17) even though past studies showed that a lack of access to sexuality-related services could put them at risk of depression and poor quality of life (14, 18). There are interrelated factors, both individual and organizational, that contribute to this situation (19, 20). The perceived lack of knowledge and skills of clinicians, as well as personal discomfort and misconceptions related to sexuality are among the most reported barriers (16, 21, 22). For example, clinicians expect clients to raise sexuality-related issues when needed (16), while clients that perceive sexuality as a taboo subject expect health professionals to address the subject first (9).

To overcome sexual difficulties, Canadian (23), American (24), and Australian (25) stroke guidelines recommend addressing sexuality during rehabilitation. A recent systematic review showed that post-stroke sexual rehabilitation interventions could significantly improve individuals' sexual functioning and sexual satisfaction (26). Moreover, considering the various factors influencing sexuality, an interdisciplinary approach to sexual rehabilitation is recommended (27, 28). The findings from a recent Australian study conducted with diverse stroke rehabilitation stakeholders (i.e., persons with stroke, partners, clinicians, and researchers) highlighted priority subjects and intervention methods that should be integrated regarding sexuality in post-stroke rehabilitation, such as strategies to resume sexual activities and communication between persons with stroke and their partner or clinicians (29). However, to our knowledge, no studies have addressed key considerations (e.g. “by who?,” “when?,” “how?”) regarding the development and application of these intervention methods, or solutions, for use in clinical practice and research. Therefore, because of the important incidence of stroke worldwide, the high prevalence of sexual difficulties post-stroke and their significant impact on the quality of life of persons with stroke, it is crucial to generate evidence-based solutions tailored to stakeholders' needs while considering barriers related to the inclusion of sexuality in rehabilitation services.

### Sexual Rehabilitation and Theories for Change of Practice

Considering the multiple barriers experienced by people with stroke, clinicians, and other stakeholders in relation to post-stroke sexual rehabilitation, it appears important to develop complex, or multifactorial, interventions that can improve the implementation of clinical practice guidelines recommendations. For example, A recent systematic review of 114 studies on inclusion of sexuality in the care of people with disability or chronic illness by McGrath et al. (20) included seven studies specifically dedicated to training of clinicians. The synthesis of these results showed that clinicians may benefit from training on sexual rehabilitation, ideally through an interdisciplinary approach, to improve their self-reported knowledge, attitude, comfort with sexuality and self-reported frequency of addressing sexuality. However, this kind of single stand-alone training can only partially address the multiple barriers influencing the provision of sexual rehabilitation, as individual and environmental factors respectively influence behavior and should be considered when planning interventions to change practice (30). Therefore, focusing only on clinicians' lack of knowledge and skills may prove to be insufficient in leading to significant change in clinical practice.

### Intervention Mapping for Improving Sexual Rehabilitation Services

This study is being conducted to address the concerns of five participating organizations regarding the lack of services related to post-stroke sexuality. Considering that changes in provision of sexual rehabilitation is complex, multiple interventions simultaneously targeting people with stroke, partners, clinicians, and clinical managers are needed to support the development and delivery of better services related to sexuality post-stroke.

To date, two studies have aimed to improve the provision of post-stroke sexual rehabilitation with a focus on changing clinicians' behaviors by creating and implementing sexuality interview guides (19, 31). The effectiveness of these implementation studies was mixed, and they did not assess the sustainability of practice changes post-implementation. This is on concordance with McGrath et al. (20) conclusion that multifactorial implementation programs were needed to lead to an effective change in the provision of sexual rehabilitation services.

Intervention Mapping is a structured process that aims to optimize a practice (e.g., non-provision vs. provision of sexual rehabilitation services) by identifying the behaviors underlying the practice and acting upon the factors (i.e., determinants) that influence the behavior (32, 33). According to a systematic review of 17 studies (34), Intervention Mapping has shown to be an effective process in guiding the development of methods and programs aimed at changing practices, such as adherence to clinical guidelines (35), assessment and treatment of risk factors (36), and provision of educational support (37). To our knowledge, Intervention Mapping has never been used to guide the development of programs and interventions designed to improve sexual rehabilitation for any health condition. In fact, very few studies have been conducted on sexual rehabilitation post-stroke, and most focused only on assessing the effects of one specific intervention method, such as pelvic floor muscle training (26). Therefore, there is a need for a complex intervention addressing the multiple challenges faced in stroke rehabilitation regarding sexuality. Because of its structured process and the evidence supporting its effectiveness in past studies, Intervention Mapping has the potential to successfully guide multiple stakeholders' needs assessment and subsequent development of a multifactorial program that targets the main determinants, ultimately improving clinical practice related to post-stroke sexual rehabilitation.

## Main Objectives

The general aim of this study is to co-design with stakeholders (namely: persons with stroke, partners, clinicians, managers, and researchers) a theory-driven program to improve post-stroke sexual rehabilitation services. The specific objectives of this study are to:

1. Describe the factors that influence the actual provision of sexual rehabilitation services in post-stroke rehabilitation from the perspective of various stakeholders.
2. Identify stakeholder's priorities and needs regarding sexual rehabilitation services and develop a preliminary theory-driven program.
3. Identify the behavior change strategies that can be used to address the prioritized factors and describe their expected action mechanisms.
4. Develop and test the behavior change strategies that will target the identified determinants.

## Materials and Methods

### Design

This qualitative study is guided by an Intervention Mapping approach (32) and co-design methods (38). This innovative combination of two approaches and methods has to our knowledge never been studied, but shows promise in leading to better results in the future. Although it is recommended to include stakeholders in each step of the Intervention Mapping approach, it does not provide clear guidelines regarding how and when to include them during the research process. Therefore, combining this approach with evidence-based co-design methods has a greater potential to lead to a deeper, more relevant, integration of stakeholders in the research process, and to develop creative and applicable strategies to improve post-stroke sexual rehabilitation services. The qualitative methods used in this study were targeted in concordance with most of the Consolidated criteria for reporting qualitative studies (COREQ) (39).

The Intervention Mapping approach (32) will orient the study process by addressing the corresponding objective [e.g., Step 1 for objective (1) through four steps: (1) needs assessment; (2) preparing matrices of change objectives; (3) selecting theory-informed intervention methods and practical strategies; and (4) producing program]. The Intervention Mapping Approach will therefore guide the process aiming to better understand stakeholders' needs and orient the creation of the matrices of change. In the latter, clear change objectives and indicators will be established to assess the upcoming program's impact and outcomes once it will be developed.

The co-design methodology has evolved in recent years, into a paradigm whereby various stakeholders work toward the same objective throughout the research process. The aim of co-design is for the entire team to focus on one challenge to better understand it and to work collaboratively toward finding creative solutions to resolve or improve it (40–43). The co-design methodology that will be used in this study follows four phases: (1) exploration; (2) co-design; (3) validation; and (4) development (38). The exploration phase aims at gaining a deeper understanding of the situation that needs to be changed by exploring the stakeholders' perspectives, experiences, and needs. The co-design phase implies the active collaboration between stakeholders and the researchers to generate innovative ideas for elaborating potential solutions that could solve the problems related to the situation. The validation phase consists of presenting the innovative ideas and potential solutions to selected experts to validate some aspects, such as their relevance and feasibility. Finally, the development phase consists of transforming the agreed upon solutions from the validation phase into concrete strategies that could be tested in the future.

To consider a variety of perspectives, persons with stroke, partners, clinicians, managers, and researchers will participate actively in the research process and/or development of the program by taking either part in an advisory group, Lego® groups, or work groups at different stages of the project. The perspectives of persons with stroke and partners will be considered by using three different strategies. First, a review of the literature regarding persons with stroke and partners' priorities and needs related to post-stroke rehabilitation and sexuality will be realized. Second, a person who received post-stroke rehabilitation and the partner of a person with stroke will be involved in the advisory group. Finally, two work groups will be conducted with post-stroke individuals and partners. Clinicians and managers will participate in the Lego® or work groups, and a subset will be included in the advisory group. Researchers will be included in the advisory group. Participation methods for each of the three groups, along with the study timeline and related deliverables, are presented in Table 1.

**Table 1:** Course of the study and related deliverables according to the co-design methodology by Morales et al. (38) and the Intervention Mapping approach (32).

Types of participants^*^	**Co-design phases**					
	**Introduction**	**Exploration**	**Co-design**	**Validation**	**Development**	Pretest^**^
Advisory Group (person with stroke, partner, clinicians, manager, researchers)	X		X		X	
Lego® groups (clinicians)		X	X			
Work groups (1) persons with stroke and partners, (2) clinicians, (3) managers)			X	X		X
Deliverables	Definition of the challenge and general objective of the program	Concrete/deep understanding of participants' needs, experiences, and potential solutions	Preliminary program, matrix of change, and choice of behavior change strategies	Improved and official program, matrices of change, and behavior change strategies	Development of preliminary behavior change interventions and complete description according to the TIDIeR checklist (44)	Improved and official selection of behavior change interventions for the program

* *Participants' characteristics and affiliations could not be provided at the time of submission of this manuscript since recruiting was ongoing*.

** *The pretest phase was separated from the development phase for clarity, although Morales et al. (38) combines them. TIDIeR, Template for Intervention Description and Replication (44)*.

### Theoretical Frameworks

We will draw from a well-known determinant framework, the Theoretical Domain Framework (TDF), and on the International Classification of Functioning, Disability and Health (ICF) core set for stroke (2) to guide data collection and analysis at each step of the study. The TDF was developed using 33 theories related to behavior change (45) and is composed of 14 distinct domains: (1) knowledge; (2) skills; (3) social/professional role and identity; (4) beliefs about capabilities; (5) optimism; (6) beliefs about consequences; (7) reinforcement; (8) intentions; (9) goals; (10) memory, attention, and decision processes; (11) environmental context and resources; (12) social influences; (13) emotions; and (14) behavioral regulation. These domains help implementation teams better understand what factors, or determinants, are likely to positively or negatively affect practices or behaviors (45). The ICF core set for stroke will be used to deepen data collection and analysis as it has been suggested that the TDF lacks exhaustivity regarding certain key domains such as environmental factors (46). Although all the factors of the ICF will be addressed in this study in conjunction with the TDF, an emphasis will be put on the environmental factors, more specifically “Products and technology,” “Natural environment and human-made changes to environment,” “Support and relationships,” “Attitudes” and “Services, systems and policies” (47). Moreover, the interrelation between different levels of environment (i.e., micro, meso, macro) will be considered (48).

### Population

Persons with stroke, partners, clinicians and managers will be recruited from five different stroke rehabilitation centers among three large cities, namely Montreal (*n* = 3), Laval (*n* = 1), and Quebec City (*n* = 1) in the province of Quebec (Canada). These rehabilitation centers offer services to people who sustained a stroke in the subacute (<3 months post-stroke) or chronic phases (>6 months post-stroke) of stroke recovery (49). More specifically, participating rehabilitation centers offer up to four types of stroke rehabilitation programs: (1) inpatient intensive rehabilitation; (2) early supported discharge; (3) outpatient rehabilitation; and (4) community-oriented rehabilitation. Each of the participating stroke rehabilitation programs offers services in an interdisciplinary approach. Only one out of the five programs has access to sexology services. To be eligible to participate in the study, persons with stroke will have to be in the chronic phase and have experienced most of the phases of post-stroke rehabilitation. It will be suggested to eligible persons with stroke that their partner participate in the study. Other potential partners that may be interested in participating in the study will have to be in a relationship with a person with a stroke in the chronic phase. Eligible researchers will have expertise in stroke rehabilitation, sexuality, implementation, or behavior change research.

### Sample and Recruitment

Persons with stroke, partners, clinicians and managers will be recruited from participating stroke rehabilitation centers. Researchers will be recruited via the research team's professional networks. An effort will be made to recruit persons with stroke that present, or partners that have an experience with, post-stroke cognitive or communication impairments. For each step of the study, different groups of participants will be required: an advisory group, two Lego® groups, and three work groups.

The advisory group will be composed of one person with stroke, one partner, four clinicians from varied disciplines, one coordinator, one manager and four researchers. The advisory group will orient the general course of the study and be actively involved in the co-design phase (i.e., creation of the preliminary program) and in the development phase (i.e., creation of the behavior change strategies).

The Lego® groups (*n* = 2) will each rally five clinicians from varied disciplines and rehabilitation centers. One group will be composed of clinicians from inpatient or early supported discharge, and the other group will include clinicians from outpatient or community-oriented rehabilitation. Therefore, for each participating rehabilitation center, one clinician corresponding to each Lego® group will be selected (total of 10 participants in Lego® groups, 2 per rehabilitation center). This division between each group is related to the substantial difference between the needs of the clientele and clinicians' respective work contexts. Participants in the Lego® groups will be recruited by convenience to optimize heterogeneity by varying the disciplines included in each of them.

The three work groups will be composed of (A) up to 20 clinicians from various disciplines and (B) up to 15 managers and coordinators, equally representing each of the participating rehabilitation centers (i.e., *n* ≤ 4 clinicians and *n* ≤ 3 managers/coordinators per rehabilitation center) and (C) up to 12 persons with stroke and partners. The work groups will take part in the validation phase (i.e., improving the preliminary program and orienting the choice of behavior change strategies) and in the pretest phase, aimed at improving the preliminary behavior change strategies. Considering their different perspectives, the three work groups will operate separately to facilitate discussion and ensure that participants are comfortable sharing information. This methodological choice is related to the pretest of the co-design methods that was conducted with a group of five-graduate students.

### Course of the Study and Co-Design Methods

The course of the study is summarized in Table 1. Although the co-design methodology (38) described above will guide the whole process of the study, the activities of the Lego® groups will be conducted using an adapted version of the Lego® Serious Play® method (50) in phase 1 (exploration).

Using Lego® as a projective method to optimize in-depth group discussions, participants in the Lego® groups will build a model representing their answer to the question asked by the facilitators. Participants will also reflect together to raise a better understanding of the challenging situation and potential solutions. Given that participants in each group will be in different locations, the Lego® Serious Play® activities will be conducted online via *Zoom Pro* and each participant will receive a Lego® Serious Play® Starter kit. This adapted online version of the Lego® Serious Play® method was pretested in the context of this study and shown to be feasible.

The three other phases (i.e., co-design, validation, and development) will be conducted using iterations between preparatory work by the research team and presentation to the group associated with each phase (advisory, Lego® of work groups) and collecting participants' feedback for improvement.

### Data Collection

Participants in every group (i.e., advisory group, Lego® groups, work groups) will provide sociodemographic information, such as age, gender, occupation, and professional experience (if applicable). Every group activity will be conducted online, recorded digitally, and co-animated by two experienced facilitators. In the Lego® groups, pictures of the Lego® models that participants will create will be stored by the research team. During group activities, the two facilitators will use guides tailored to each activity's specific purpose (see deliverables in Table 1). The facilitators for group activities will be trained to use the TDF and will keep a list of the 14 domains so that they make adjustments in real time; this will also require ongoing analysis to ensure each domain is adequately explored. The recordings of every group activity will be transcribed verbatim. Verbatim and pictures will be transferred into the NVivo software for analysis. Facilitators of group activities will write their perceptions and reflections in a logbook during data collection and analysis.

### Data Analysis

The verbatim from each group activity will be analyzed by two independent evaluators using semi-deductive thematic analysis (51). First, multiple readings of the verbatim will be done to obtain a better understanding of the content. Each verbatim and pictures will be coded using a preliminary coding scheme based upon the TDF domains and the ICF core set for stroke (2). Moreover, additional codes (2) directly related to verbatim's content could be added to be as representative as possible of the discussion. The codes will then be organized by categories, themes, and sub-themes when needed using semi-deductive analysis (51). Moreover, data will be triangulated between (1) group activities' verbatim, (2) pictures (when applicable), and (3) the team's logbook. The data analysis will highlight the most dominant factors from each of the group activity. Consistent with the co-design paradigm, the advisory and work groups will be consulted when needed. The choice of behavior change strategies will be oriented using the Behavior Change Wheel (30), and a theory-based taxonomy will be used to guide the detailed development of each strategy (52). Moreover, the development and description of the concrete application of each strategy will be realized according to the Template for Intervention Description and Replication (TIDIeR) scale (44), which will promote the exhaustive consideration and description of relevant elements (e.g., level of environment, stakeholders, material) for an optimal application of the strategies and future replication.

### Ethical Considerations

This study was approved (#MP-50-2022-1277) by the research ethics board of the Centre for Interdisciplinary Research in Rehabilitation of Greater Montreal (CRIR). To be included in the study, each potential participant will have to read and sign a consent form. Participants will be free to withdraw from the study at any time.

## Discussion

This study will be conducted in collaboration with five Canadian post-stroke rehabilitation centres, using innovative methods combining the Intervention Mapping approach, co-design methodologies, and a rigorous theoretical framework. First, these methods will allow for a better understanding of the challenging situation regarding the lack of post-stroke sexual rehabilitation, according to the experience of persons with stroke, partners, clinicians, managers, and researchers. Secondly, these methods will orient the production of a multifactorial program with tools and strategies aiming to improve sexual rehabilitation. The program will likely be more relevant and useful for persons with stroke, partners, clinicians, managers, and organizations since stakeholders will actively participate in each phase of the study (53). We believe that the methods presented in this paper may not only be useful for guiding rehabilitation practice among a stroke population but could also provide valuable insights to improve rehabilitation services delivered to other clienteles. Finally, by involving various stakeholders in using innovative and participatory approaches underpinned by robust theoretical frameworks, this study can contribute to implementation science (54).

### Strengths and Limitations

This protocol is characterized by numerous methodological strengths. First, the data collection methods using an adapted version Lego® Serious Play® method has been successfully pretested with five postgraduate students in preparation of this study. Secondly, by combining in-depth (i.e., adapted version of the Lego® Serious Play® method) and exhaustive (i.e., group discussions and collaborative reflections and work) data collection methods with each relevant group of stakeholders, namely persons with stroke, partners, clinicians and managers, results will be more likely to provide an accurate representation of participant's perceptions and experiences. Finally, the methods were chosen in concordance with the COREQ (39) and answer to the scientificity criteria in qualitative research (55). In fact, the analysis informed by robust frameworks, triangulation of data, and the ongoing analysis validation with the research team and the advisory and work groups will enhance the credibility and reliability of the results (55). Moreover, the variety of participants in the sample regarding stakeholders' type (e.g., persons with stroke, clinician) and location (i.e., three different regions in Quebec, Canada) will promote transferability of our results to other stroke rehabilitation settings.

The methods included in this protocol also have certain limitations. First, the sample of participants included in this qualitative study could limit the transferability of the results as they may not be representative of all the stakeholders in their category. In fact, since participants will be recruited in stroke rehabilitation settings, results are likely to be contextualized to this specific reality and may not be representative of stakeholders from other contexts of care, such as acute settings and community-based services. This could especially limit the transferability of results regarding the experiences, priorities and needs of persons with stroke and partners. However, a thorough consideration of the literature regarding people with stroke and their partners will be used in the assessment of needs and the program's development to complement the participant's contribution in each step of the study. Moreover, the inclusion of five different stroke rehabilitation centers from various geographic locations and various disciplines of clinicians will mitigate the impact of specific organizational or professional cultures. Nevertheless, the results will be contextualized to Quebec's healthcare system context and the stroke population. Therefore, results will need to be interpreted with caution in the context of other settings or populations. Secondly, the fact that data collection will be realized remotely may hinder participant's involvement during activities and limit the depth of data collected. However, the presence of trained facilitators in every group and the variety of data collection methods will foster participant's involvement and ensure rich and in-depth discussions during data collection.

## Data Availability Statement

The original contributions presented in the study are included in the article/supplementary material, further inquiries can be directed to the corresponding author.

## Ethics Statement

The studies involving human participants were reviewed and approved by the Research Ethics Committee of the Centre for Interdisciplinary Research in Rehabilitation of Greater Montreal (CRIR). Written informed consent was not provided because this manuscript is a protocol for a study that was not started yet. Therefore, no participants were recruited at the time of the submission of this article. In the future, when we will recruit participants, each of them will have to provide consent by signing an information and consent form. The recruitment of participants has been initiated.

## Author Contributions

L-PA was primarily responsible for writing all of the sections of the article. DA, EM, AT, JF, BV, and AR have contributed to the development of the methods that will be used in the research and have revised and improved the manuscript. All authors contributed to the article and approved the submitted version.

## Funding

This research project was supported by funding from the Centre for Interdisciplinary Research in Rehabilitation of Greater Montreal (CRIR). The authors gratefully acknowledge that L-PA was supported by doctoral scholarships from the Canadian Institutes for Health Research, the Fonds de recherche du Québec en santé (FRQS), the School of Rehabilitation of the Université de Montréal (UdeM), the Canadian Occupational Therapy Foundation (COTF), and the Ordre des ergothérapeutes du Québec (OEQ). DA was supported by doctoral scholarships from the Mission Universitaire de Tunisie, the School of Rehabilitation of UdeM, the graduate and postdoctoral studies of UdeM, the CRIR, and the Fonds Wilrose Desrosiers et Pauline Dunn. AT was supported by a career award from the FRQS.

## Conflict of Interest

The authors declare that the research was conducted in the absence of any commercial or financial relationships that could be construed as a potential conflict of interest.

## Publisher's Note

All claims expressed in this article are solely those of the authors and do not necessarily represent those of their affiliated organizations, or those of the publisher, the editors and the reviewers. Any product that may be evaluated in this article, or claim that may be made by its manufacturer, is not guaranteed or endorsed by the publisher.
